# Mitomycin C in combination with radiotherapy as a potent inhibitor of tumour cell repopulation in a human squamous cell carcinoma

**DOI:** 10.1038/sj.bjc.6600081

**Published:** 2002-02-01

**Authors:** W Budach, F Paulsen, S Welz, J Classen, H Scheithauer, P Marini, C Belka, M Bamberg

**Affiliations:** Department of Radiation Oncology, University of Tuebingen, Hoppe-Seyler-Str. 3, D-72076 Tuebingen, Germany

**Keywords:** Mitomycin C, radiotherapy, repopulation, xenograft, squamous cell carcinoma

## Abstract

The potential of Mitomycin C in combination with fractionated irradiation to inhibit tumour cell repopulation of a fast growing squamous cell carcinoma after fractionated radiotherapy was investigated *in vivo*. A rapidly growing human squamous cell carcinoma (FaDu_dd_) was used for the study. For experiments, NMRI (nu/nu) mice with subcutaneously growing tumours were randomly allocated to no treatment, Mitomycin C, fractionated irradiation (ambient: 11x4.5 Gy in 15 days), or fractionated irradiation combined with Mitomycin C. Graded top up doses (clamped blood flow: 0–57 Gy) were given at day 16, 23, 30 or 37. End point of the study was the time to local tumour progression. Data were examined by multiple regression analysis (Cox). Mitomycin C alone resulted in a median time to local tumour progression of 23 (95% confidence limits: 17–43) days, fractionated irradiation in 31 (25–35) days and combined Mitomycin C plus fractionated irradiation in 65 (58–73) days (*P*=0.02). Mitomycin C decreased the relative risk of local recurrence by 94% (*P*<<0.001) equivalent to 31.7 Gy top up dose. Repopulation accounted for 1.33 (0.95–1.72) Gy per day top up dose after fractionated irradiation alone and for 0.68 (0.13–1.22) Gy per day after fractionated irradiation+Mitomycin C (*P*=0.018). Mitomycin C significantly reduces the risk of local recurrence and inhibits tumour cell repopulation in combination with fractionated irradiation *in vivo* in the tested tumour model.

*British Journal of Cancer* (2002) **86**, 470–476. DOI: 10.1038/sj/bjc/6600081 www.bjcancer.com

© 2002 The Cancer Research Campaign

## 

Repopulation of surviving clonogenic tumour cells during fractionated radiotherapy has been identified as an important factor associated with clinical failure in squamous cell carcinoma of the head and neck (Withers *et al*, 1988; Fowler and Lindstrom, 1992). According to the results of randomized clinical trials (Dische *et al*, 1997; Dobrowsky and Naude, 2000) the dose needed to counteract tumour cell repopulation accounts for approximately 0.4 Gy per day longer overall treatment time. In experimental human tumour models, it was shown that the repopulation rate is tumour cell line dependent, varying over a wide range between 0.5–4.6 Gy per day (Trott, 1990; Baumann *et al*, 1994; Budach *et al*, 1997). Moderately and excessive accelerated radiation schedules have been employed to overcome resistance induced by tumour cell repopulation. These studies gave evidence that one can decrease the total radiation dose in accelerated radiation schedules without compromising local tumour control (Dische *et al*, 1997; Dobrowsky and Naude, 2000). An improvement of local tumour control was observed only in studies that did not decrease the total radiation dose in the accelerated arm. However, in the latter studies acute and late radiation toxicity was also significantly enhanced in the accelerated treatment arms, indicating that an unambiguous therapeutic benefit for accelerated treatment schedules in head and neck cancer has not been proven (Beck-Bornholdt *et al*, 1997; Haffty *et al*, 1997; Horiot *et al*, 1997).

Squamous cell carcinomas of the head and neck have been shown to be a chemosensitive disease. Cisplatin and taxane based regimens in combination with 5-fluorouracil render overall response rates of 80–90% in locally advanced disease (Khuri *et al*, 2000). In spite of this high efficacy, induction chemotherapy before definitive radiation therapy was unable to improve the patients' outcome (Rosenthal *et al*, 1994). However, simultaneous or alternating chemo-radiotherapy has been shown to improve locoregional tumour control and survival compared to radiotherapy alone (Pignon *et al*, 2000) no matter whether conventional or hyperfractionated – accelerated radiation schedules were used (Merlano *et al*, 1996; Brizel *et al*, 1998; Calais *et al*, 1999; Jeremic *et al*, 2000). The reason for this discrepancy in the clinical findings is not well understood. It has been speculated that induction chemotherapy kills preferentially well oxygenated tumour cells leaving a smaller but more hypoxic and more radioresistant tumour at the start of radiotherapy (Rosenthal *et al*, 1994). Another conjecture is that clonogenic tumour cells surviving the induction chemotherapy exhibit accelerated repopulation already before or at the beginning of radiation therapy, whereas with radiotherapy alone, accelerated repopulation is thought to start not before week 3 or 4 of the radiation series.

According to the results of a large set of experimental data, the benefit of simultaneous chemo-radiation is regarded to result mainly from radiosensitization of tumour cells, additive tumour cell kill, and, for certain drugs like Mitomycin C (MMC), the killing of hypoxic tumour cells (Rockwell, 1982). The impact of another potentially important mechanism that may also be responsible for the beneficial effect of simultaneous chemo-radiation, the inhibition of repopulation during the 5–7 week course of radiation therapy by cytostatic drugs, has not been systematically investigated. We, therefore, designed a set of experiments using a rapidly growing human head and neck squamous cell carcinoma to test the hypothesis whether inhibition of repopulation is an important mechanism of action for simultaneous chemo-radiation.

## MATERIALS AND METHODS

### Tumour cell line

The human hypopharyngeal squamous cell carcinoma cell line FaDu_dd_ was used for the study. The cell line was kindly provided by M Baumann (Dresden, Germany). FaDu_dd_ is a well characterized subline derived from the ATCC cell line FaDu. The DNA content is aneuploid and the p53 tumour suppressor gene is mutated. FaDu_dd_ exhibits rapid growth *in vitro* and *in vivo* on nude or SCID mice (Baumann *et al*, 1994; Budach *et al*, 1997). The cell line is only weakly immunogenic on nude mice. Former experiments had shown that 6 mm xenografts of FaDu_dd_ on nude mice were not responsive to 5-fluorouracil and cisplatin at maximally tolerated dose (MTD) (Classen *et al*, 1998), but exhibited response to MMC (at MTD).

### Experimental animals

Immunodeficient NMRI-(nu/nu)-nude mice were bred in a specific pathogen free animal colony at the University of Essen. At an age of 4–6 weeks, animals were brought to Tuebingen University and housed in an individually ventilated cage rack system (Techniplast, Italy). They were fed sterile high-calorie laboratory food and drank water supplemented by chlorotetracycline and potassium sorbate acidified to a pH of 3.0 with hydrochloric acid *ad libitum*. All animal experiments were performed in accordance with the standards required by the United Kingdom Co-ordinating Committee on Cancer Research (UKCCCR) Guidelines (1998).

### Transplantation and experimental design

**Figure 1:**
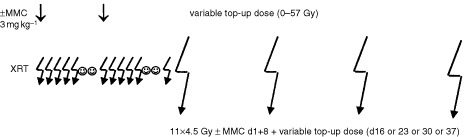
Experimental design. XRT=radiation therapy; small jagged arrows=single fractions; large jagged arrows=graded top up doses (one top up dose per animal); straight arrow=application of MMC; smiling face=weekend days without therapy; d=day.

For the experiments a source tumour was excised and tumour chunks of about 2 mm diameter were implanted subcutaneously into the right hind limb of 6–10-week old animals. Wound closure was not necessary. Approximately 2–3 weeks after transplantation visible tumour growth occurred. The tumour size was scored three times a week. When the tumours reached a volume of approximately 120 mm^3^ the animals were randomly allocated to the following treatment arms (Figure 1

): (a) MMC at day 1 and 8 at a dosage of 3 mg kg^−1^ body weight intraperitoneally (i.p.) (*n*=11); (b) fractionated radiotherapy (11×4.5 Gy in 15 days) followed by graded top up doses (0–57 Gy) at day 16, 23, 30 or 37 (one top up dose per animal) (*n*=117); (c) fractionated radiotherapy (11×4.5 Gy in 15 days) in combination with MMC on day 1 and 8 (3 mg kg^−1^ i.p., 1 h before irradiation) followed by graded top up doses (0–37 Gy) at day 16, 23, 30 or 37 (one top up dose per animal) (*n*=139); (d) no treatment (control animals) (*n*=25).

Experiments were performed in two runs. The first run consisted of 144 and the second run of 148 randomized animals. The median tumour volume at the start of treatment was 126 mm^3^ (standard deviation 39.1 mm^3^). According to the results of previous experiments (Paulsen *et al*, 1998) a single dose of 4.5 Gy was chosen during fractionated radiotherapy to meet two experimental requirements: (a) The tumours had to be smaller than 1500 mm^3^ at the time of the last top up dose (day 37). At larger sizes, tumours tend to spread to the trunk of the mice and cannot be sufficiently irradiated to achieve long term local tumour control; (b) Long term local tumour control must not be attained by fractionated radiotherapy alone. Top up doses with graded doses were administered at different intervals after fractionated radiotherapy in order to estimate isoeffective dose levels and calculate the dose needed to counteract repopulation during the interval between fractionated radiotherapy and top up irradiation (with and without MMC).

### Irradiation

A linear accelerator (6 MV photons, about 400 cGy min^−1^ dose rate) with an experimental device as described earlier (Stüben *et al*, 1994) was used. Shieldings reduced the dose to the animal body to less than 3% of the prescribed tumour dose. Ten mice were irradiated simultaneously in general anaesthesia. For the fractionated irradiation an inhalation anaesthesia with enflurane and, for the top up doses, a combination of 10 mg kg^−1^ body weight xylazinehydrochloride and 15 mg kg^−1^ body weight ketaminehydrochloride was given intraperitoneally. Radiotherapy was delivered under ambient conditions during fractionation. For the top up doses the blood flow to the tumour bearing leg was clamped at least 5 min before administration of irradiation in order to achieve an acute hypoxia.

### Chemotherapy

Animals randomized to chemotherapy received 3 mg kg^−1^ body weight Mitomycin C (Medac, Germany) intraperitoneally approximatley 1 h before radiation therapy on days 1 and 8 of the treatment. The injected volume was 0.1 ml per 10 g body weight. Animals randomized to radiotherapy alone got no injections.

### Evaluation of tumour response and follow up

Tumour size was measured with calipers in two perpendicular diameters. The tumour volume (V) was calculated as V=(a×b^2^)/2, where a and b are the long axis and the short axis, respectively. Scoring of tumour sizes took place three times per week before start of treatment and twice per week after the start of the treatment. The investigators were not blinded as to the treatment that the animals had received, when they made their measurements. Follow up was terminated at day 280 or in case of intercurrent death or if recurrent tumours after top up irradiation had grown to eight-times the initial tumour volume at the start of treatment. Body weight was recorded once a week. A modified growth delay end point, time to local tumour progression (TTP), was end point of the study. For animals randomized to receive MMC only or fractionated radiotherapy alone without top up doses, the TTP was defined as interval between the day of start of treatment to the day when the regrowing tumours reached twice the initial tumour volume. For animals randomized for a top up irradiation, the TTP was defined as interval between the day of start of treatment to the day after top up irradiation when the regrowing tumours reached twice the initial tumour volume.

### Statistical analysis

The median TTP was calculated according to the method of Kaplan and Meier (1958). Differences between groups were tested for significance by the log-rank test. The data of all animals that had received fractionated radiotherapy (±MMC, ±top up irradiation) were the basis for a multivariate analysis (Cox model) (*n*=256). Tested parameters were top up dose (0–57 Gy), MMC (yes or no), time of top up radiotherapy (0–3 weeks after fractionated radiotherapy), and MMC×(time of top up radiotherapy). The relative risk ratios derived from the Cox model were the basis for calculation of the radiotherapy top up dose equivalents for the effect of MMC and time effect with and without MMC.

## RESULTS

Acute toxicity as measured as maximal weight loss of the animals during the fractionated radiotherapy (±MMC) was only moderate. Mice receiving radiotherapy alone and radiotherapy in combination with MMC exhibited a maximal median weight loss of 10% (s.d.±6%) and 6% (s.d.±5%), respectively (not significant). The nadir of weight loss occurred at day 9 in the radiotherapy alone arm of the study and at day 8 in the radiotherapy+MMC arm of the study.

**Figure 2:**
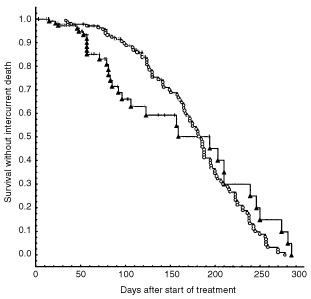
Life span of animals with locally controlled tumours after fractionated radiotherapy (11×4.5 Gy)+top up irradiation±MMC (*n*=256). Open circles indicate radiotherapy+MMC, closed triangles radiotherapy alone, and tick marks censoring for local recurrence (difference not significant).

During longer follow-up after fractionated radiotherapy intercurrent deaths occurred as a result of the limited life span of nude mice. However, the frequency and time of intercurrent deaths did not significantly differ between animals treated with and without MMC (Figure 2

).

MMC alone exhibited moderate tumour activity resulting in a median TTP of 23.0 (95% confidence limits (CL) 17.0–42.8) days (*n*=11) compared to 4.5 (CL: 3.2–5.6) days (*n*=25) in untreated control animals (*P*<0.001). After fractionated radiotherapy alone (without top up dose) a median TTP of 31.0 (CL: 25.0–34.9) days was observed compared to 64.9 (CL: 57.9–72.8) days after fractionated radiotherapy in combination with MMC (*P*=0.02). The difference in TTP after MMC alone (23.0 days) and fractionated radiotherapy alone (31.0 days) was not significant.

**Figure 3:**
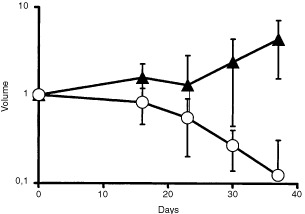
Mean tumour volume (V) at the time of top up irradiations in relation to the initial tumour volume (V0) at the start of treatment (volume=V/V0). Triangles indicate radiation therapy alone and circles radiation therapy in combination with MMC. Error bars represent the s.d. of the mean.

No major tumour regressions were observed during the fractionated radiotherapy with either treatment. However, during the interval between the end of fractionated radiotherapy and the last top up irradiation at day 37, MMC treated tumours showed a continuous and substantial decrease in tumour volume, whereas after radiotherapy alone only minor tumour regressions were observed and average tumour volumes tended to increase again after day 23 (Figure 3

).

**Figure 4:**
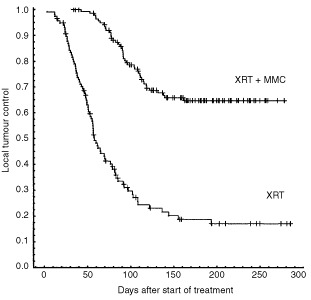
Actuarial local tumour control after fractionated radiotherapy alone+top up irradiation (lower curve, *n*=117), and fractionated radiotherapy in combination with MMC+top up irradiation (upper curve, *n*=139) (log rank test: *P*<<0.001). Tick marks represent censored cases due to intercurrent death of animals. The median top up dose in the radiotherapy+MMC group was 15 Gy compared to 30 Gy in the radiotherapy alone group.

A total of 256 animals received fractionated radiotherapy±MMC with or without top up irradiation. Locally recurrent tumours were observed in 84 out of 117 animals (71.8%) in the radiotherapy alone arm (all top up days) and in 44 out of 139 animals (31.7%) in the radiotherapy+MMC arm (all top up days) of the study (*P*<0.001), although the median top up doses were 15 Gy lower in the MMC arm (30 Gy *vs* 15 Gy) (Figure 4

). At day 150 after the start of treatment two-thirds of the animals without local recurrence were still alive (Figure 2) and 82 of 84 recurrences in the radiotherapy arm and 43 of 44 in the radiotherapy+MMC arm occurred before day 150 (Figure 4), indicating that the life span and follow up of the animals was sufficient to calculate ultimate local tumour control.

Figure 5

**Figure 5:**
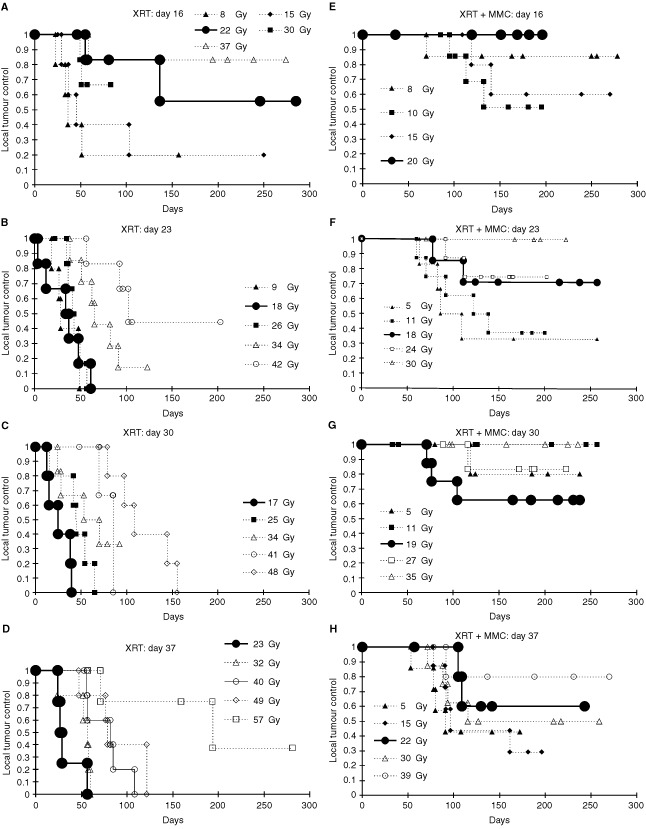
Local tumour control for individual animals depending on the top up dose and top up day. (**A**–**D**) radiotherapy alone. (**E**–**F**) radiotherapy+MMC. The displayed dose levels represent top up doses. All animals received in addition 49.5 Gy fractionated radiotherapy. The bold closed circles indicate the results for top up doses at a similar dose range (17–23 Gy) in the respective group.

displays the local tumour control data of all 256 animals that had received fractionated radiotherapy. A distinct loss of local tumour control with an increasing interval between fractionated radiotherapy and top up irradiation is obvious as well for tumours treated with radiotherapy alone as for tumours treated with radiotherapy+MMC. However, the loss of local tumour control was less pronounced in the MMC arm of the study.

The results of the multivariate analysis of TTP is shown in Table 1

**Table 1:**
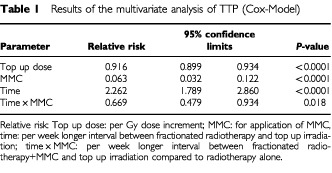
Results of the multivariate analysis of TTP (Cox-Model)

. All parameters included in the model were significant. The relative risk to develop a local recurrence was reduced by 94% in animals that had received MMC and decreased by 8% per Gy top up dose. The risk of local recurrence increased 2.3-fold per week longer between intervals of fractionated radiotherapy and top up irradiation. This risk was significantly decreased by MMC (relative risk 0.67). Based on the relative risk ratios, the effect of MMC was equivalent to 31.7 Gy top up dose. The dose required to counteract repopulation was 9.3 Gy per week (1.3 Gy per day) in the radiotherapy alone arm (Table 2

**Table 2:**
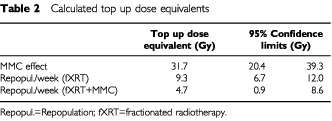
Calculated top up dose equivalents

) and 4.7 Gy per week (0.7 Gy per day) in the radiotherapy+MMC arm of the study (*P*=0.018).

**Figure 6:**
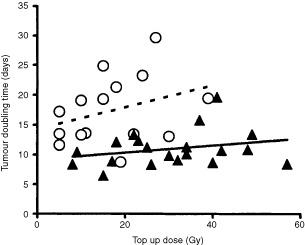
Tumour doubling times of locally recurrent tumours depending on the top up dose: Triangles indicate fractionated radiation (fRT) and circles represent fRT in combination with MMC.

Recurrent tumours after top up irradiation±MMC grew significantly slower (mean tumour doubling time (TDT): 13.7 days, 95% CL: 11.9–15.6 days) compared to untreated control tumours (mean TDT: 4.5 days, 95% CL: 3.8–5.1 days). TDT of recurrent tumours that had received MMC during fractionated radiotherapy were significantly longer (17.4 days (95% CL: 14.3–20.6 days)) than TDT of recurrent tumours that had not received MMC (11.0 days (95% CL: 9.6–12.4 days)) (*P*<0.001, Mann–Whitney *U*-test). A non-significant trend towards longer TDT of recurrent tumours were observed with increasing top up doses especially after treatment with MMC (Figure 6

).

## DISCUSSION

MMC at maximally tolerated dose in combination with fractionated radiotherapy reduced the relative risk of local recurrence by more than 90% (Table 1). This effect of MMC was equivalent to 31.7 Gy top up dose. According to former experiments (Budach *et al*, 1993) and the presented results an additive effect of MMC and radiotherapy can be assumed.

The relative risk for local recurrence increased almost 30-fold during the 3-week interval between fractionated radiotherapy and top up irradiation in animals that had not received MMC, but only 3.5-fold in animals that had received MMC (*P*=0.018). Repopulation in the interval between fractionated radiotherapy and top up irradiation accounted for 1.33 Gy per day top up dose after fractionated radiotherapy alone and for 0.68 Gy per day after fractionated radiotherapy in combination with MMC (Table 2). Inhibition of repopulation of surviving clonogenic tumour cells by MMC during this interval is the only straightforward explanation for this observation. The repopulation rate was approximately cut half by MMC. Differences in the hypoxic tumour cell fraction at the time of top up irradiation cannot serve as an alternative explanation for this observation, because the blood flow to the tumour bearing legs was clamped before top up irradiation rendering all tumours acutely hypoxic. However, the chronically hypoxic tumour cell fraction was likely larger in tumours treated with radiotherapy alone than in tumours receiving MMC and irradiation, since the latter tumours showed constant shrinkage in the interval between fractionated radiotherapy and top up irradiation and were considerably smaller at the time of top up irradiation (Figure 3). A more pronounced radiation resistance of chronically hypoxic tumour cells compared to acutely hypoxic tumour cells would also be compatible with the observations. However, experimental data do indicate the opposite, a less pronounced radiation resistance of chronically hypoxic tumour cells (Denekamp and Dasu, 1999). The inhibitory effect of MMC on repopulation is further substantiated by the observation that recurrent tumours after radiotherapy in combination with MMC grew significantly slower compared to recurrent tumours after radiation treatment alone (Figure 6).

The experimental design allowed to verify the inhibition of repopulation by MMC between day 16 and 37 after fractionated radiotherapy. Although no direct estimate of repopulation during fractionated radiotherapy can be derived from the experiments, it appears to be reasonable to assume that MMC inhibited repopulation also during the fractionated radiotherapy. Based on this assumption and presuming a constant inhibitory effect of MMC from the start of fractionated radiotherapy to the last top up irradiation, the inhibition of repopulation would account for approximately 9.2 Gy top up dose or 30% of the total MMC effect at the end of the fractionated radiotherapy (day 16) and for 23 Gy top up dose or for 50% of the MMC effect at the last top up irradiation (day 37). Translated to a 6–7 weeks clinical radiation series in head and neck cancer, this would mean that inhibition of repopulation by MMC (and potentially other cytostatic drugs) might be of major importance for the observed beneficial effects of simultaneous chemo-radiotherapy. This would only be true, if repopulation is inhibited more in the tumour than in normal tissues. A significant inhibition of repopulation in normal tissues by MMC should be clinically detectable by an enhanced radiation mucositis and dermatitis. However, in clinical trials on head and neck cancer using MMC, only little or no evidence of enhanced mucosal or dermal side effects was found, whereas locoregional tumour control was considerably increased (Haffty *et al*, 1997; Dobrowsky and Naude, 2000; Budach *et al*, 2001). This observation gives indirect evidence that repopulation of normal tissues is clinically not significantly inhibited.

Direct comparable data have, at least to the knowledge of the authors, not been published before. Repopulation of FaDu_dd_ tumours during fractionated radiotherapy alone has been intensively studied by Baumann and colleagues (Baumann *et al*, 1994; Schoene *et al*, 2001). They reported a repopulation rate equivalent to 1.0–1.5 Gy per day for ambient conditions, which compares well to the present findings (1.3 Gy) and to earlier *in vitro* results of our group (Budach *et al*, 1997). In contrast to Baumann *et al* (1994), radiotherapy (top up) in the present study was administered under acutely hypoxic conditions. Under these conditions, cells are up to three-fold more radiation resistant compared to oxygenated cells. Therefore, larger repopulation rates (as measured in Gy per day) could have been expected in our experiments. However, according to the data of Baumann *et al* (2001), the hypoxic fraction of surviving clonogenic tumour cells at the end of the fractionated radiotherapy is close to 100% in FaDu_dd_ tumours on nude mice. These data gave indirect evidence that top up irradiations under ambient conditions might have given identical results and that our results are entirely consistent with results of Baumann *et al* (1994, 2001).

The dose needed to counteract tumour cell repopulation can be calculated from the CHART (Dische *et al*, 1997) and V-CHART (Dobrowsky and Naude, 2000) clinical trials. According to these trials repopulation without chemotherapy accounts for approximately 0.4 Gy per day, which is substantially lower than in the tested experimental system, suggesting that the potential clinical benefit derived from the inhibition of repopulation by chemotherapy during radiotherapy might be overestimated by our findings. The impact of repopulation during simultaneous chemo-radiation has not been measured in clinical studies. However, indirect evidence that chemotherapy inhibits repopulation comes from the study of Merlano *et al* (1996). They randomized patients with inoperable head and neck cancer to receive either radiotherapy alone with 70 Gy in 7 weeks or alternating chemo-radiation consisting of four cycles of cisplatin and 5-fluorouracil given every third week for 5 days and radiotherapy (60 Gy) given in three courses of 20 Gy in the intervals between chemotherapy cycles. In spite of 15% lower total dose and 1 week longer overall treatment time in the radiation series of the chemo-radiation arm, an absolute survival benefit of 14% (*P*<0.01) was observed for the chemo-radiation arm of the study. The extent of the observed survival benefit is not smaller than in other chemo-radiation trials using identical overall treatment times or accelerated treatments in both study arms (Brizel *et al*, 1998; Calais *et al*, 1999; Dobrowsky and Naude, 2000; Jeremic *et al*, 2000). The question arises whether accelerated radiation schedules are necessary, when simultaneous chemo-radiation is used. If repopulation is inhibited by chemotherapy in the majority of tumours, the answer would be no. Clinical trials are required to test this hypothesis.

The mechanism behind the observed inhibition of repopulation by MMC was not subject of the current study. MMC is known to induce a marked cell cycle arrest in the G2/M phase (Franchitto *et al*, 1998; Heinrich *et al*, 1998; Sugiyama *et al*, 2000). The duration of this cell cycle arrest has not been well documented, but is unlikely to persist for several weeks as would be required to explain the duration of inhibition of repopulation in our experiments. Short-term exposure (2.5 min) to MMC of human Tenon's fibroblasts has been shown to suppress cell proliferation for at least 6 weeks (Woo *et al*, 1997). However, no data on long-term changes in the cell cycle distribution or expression of cyclins after MMC are available. The mechanism of the inhibitory effect of MMC on repopulation remains elusive and will be subject of subsequent investigations.

Although we found evidence that chemotherapy can inhibit repopulation, some limitations of the studies have to be kept in mind. Only one tumour cell line was investigated with one cytostatic drug (MMC) so that we do not know whether our observations will be typical for other tumour cell lines and cytostatic drugs. Large differences in the sensitivity of human tumours towards MMC have been observed. The tumour response is influenced by the reductive enzyme profile of the tumour (Gan *et al*, 2001). A rapid MMC metabolism is associated with a pronounced tumour response (Phillips *et al*, 2000) as was observed in the investigated tumour cell line. Therefore, the beneficial effects of MMC in combination with radiotherapy may not be as pronounced in tumour cell lines that are poor metabolizers of MMC. The data did not allow to evaluate whether the repopulation dynamics was any different comparing the first and the last week of the observation period or was different during fractionated radiotherapy. The hypoxic tumour cell fraction was not assessed at any time during the experiments. Therefore one has to be cautious to generalize the results and conclusions for clinical practice cannot be drawn.

But despite these critical appointments we demonstrated, as a proof of principle that chemotherapy, especially MMC, is able to inhibit tumour cell repopulation significantly. Inhibition of repopulation appears to be a potentially important mechanism for the beneficial effects of simultaneous chemo-radiation. An accelerated radiotherapy might not be necessary if an effective chemotherapy is used concurrently. This issue deserves further experimental and clinical investigations.
